# Fracture of the Pelvis—Laceration of the Bladder—Recovery

**Published:** 1843-08

**Authors:** 


*Fracture of the Pelvis—Laceration of the Bladder—Recovery.*—A patient with the above accident recently fell under the care of Dr. Walker, of Charlestown. The patient was precipitated from a rail-road, with engine, cars, &c., into a river. On examination, crepitus was found to exist in the bones of the pelvis, following any attempt at motion of these parts. There was a tumor in right iliac region, extending from Poupart's ligament almost to umbilicus, and situated between peritoneum and abdominal muscles, excessively tender, and when handled followed by sickness and vomiting. The bladder was full at the time of the accident. A catheter, when introduced into the urethra, on passing under the arch of the pubes, turned to the right side, upwards and outwards, and having passed some distance in that direction discharged six ounces of bloody urine, which was coincident with the subsidence of the tumor. Pulse small, and at times almost imperceptible, attended with jactitation, cool skin and some sweating. Catheter was passed three times in twelve hours, with relief. Sense of tumor in pelvis, but no disposition to pass urine. To procure free exit and prevent distension from accumulation of urine, the bladder was opened much as in the operation for lithotomy, and constant evacuation maintained through orifice. From the time of the operation there was no more vomiting, and sickness soon ceased; the unfavorable symptoms disappeared, and perfect comfort ensued. No sloughing of parts about lacerated portion. The patient continued to do well, and on the 25th day consolidation of fractured bones had taken place, and he has since been able to go out.—*Ibid.*

